# Continuous Optical Zoom Compound Eye Imaging Using Alvarez Lenses Actuated by Dielectric Elastomers

**DOI:** 10.3390/biomimetics9060374

**Published:** 2024-06-20

**Authors:** Chuanxun Chen, Qun Hao, Lin Liu, Jie Cao, Zhibo Qiao, Yang Cheng

**Affiliations:** 1 Key Laboratory of Biomimetic Robots and Systems, Ministry of Education, Beijing Institute of Technology, Beijing 100081, China; 3220205080@bit.edu.cn (C.C.); qhao@bit.edu.cn (Q.H.); 3220225097@bit.edu.cn (L.L.); caojie@bit.edu.cn (J.C.); 3220230613@bit.edu.cn (Z.Q.); 2 Yangtze Delta Region Academy of Beijing Institute of Technology, Jiaxing 314003, China; 3 National Key Laboratory on Near-Surface Detection, Beijing 100072, China

**Keywords:** compound eye, continuous optical zoom, Alvarez lens, dielectric elastomer

## Abstract

The compound eye is a natural multi-aperture optical imaging system. In this paper, a continuous optical zoom compound eye imaging system based on Alvarez lenses is proposed. The main optical imaging part of the proposed system consists of a curved Alvarez lens array (CALA) and two Alvarez lenses. The movement of the CALA and two Alvarez lenses perpendicular to the optical axis is realized by the actuation of the dielectric elastomers (DEs). By adjusting the focal length of the CALA and the two Alvarez lenses, the proposed system can realize continuous zoom imaging without any mechanical movement vertically to the optical axis. The experimental results show that the paraxial magnification of the target can range from ∼0.30× to ∼0.9×. The overall dimensions of the optical imaging part are 54 mm × 36 mm ×60 mm (L × W × H). The response time is 180 ms. The imaging resolution can reach up to 50 lp/mm during the optical zoom process. The proposed continuous optical zoom compound eye imaging system has potential applications in various fields, including large field of view imaging, medical diagnostics, machine vision, and distance detection.

## 1. Introduction

The compound eyes of insects are composed of thousands of ommatidia that are closely arranged on a curved surface. Such a unique structure endows insects with a compact structure, a wide field of view (FOV), and sensitivity to moving objects [1,2,3,4,5]. In recent years, triggered by the advantages of compound eyes, many studies of the imaging system have been devoted to mimicking the natural compound eye. In 2016, an imaging system inspired by the superposition compound eye and the retinal structure of elephant nose fish was proposed, which contained 2304 ommatidia and achieved an optical magnification of 4× [6]. In 2020, a bionic-compound-eye structure with an imaging FOV of 28° × 22° was proposed, which contained 2400 ommatidia with a focal length of 10 mm and a fixed work distance of 7.8 mm [7]. In 2022, a wide-band spherical compound eye camera that can achieve a FOV of 360° × 171° was proposed. It contains 234 sub-eyes with a diameter of 6.35 mm and a focal length of 10 mm [8]. Although these artificial compound eye imaging systems have excellent imaging performance, the focal length of the ommatidia lacks adjustability, and these imaging systems do not permit tunable imaging, which significantly limits the practical applications, for instance, distinguishing objects at different distances. In general, it is plausible to expect that an imaging system incorporating the large FOV feature of the insect compound eye and the tunable focal length ability of the human eye may simultaneously offer a large FOV, as well as varifocal capabilities for practical imaging applications. Fortunately, several attempts have been recently made to develop dynamically tunable artificial compound eye imaging systems using an adaptive lens array, i.e., a single lens with a tunable focal length. Wei et al. have demonstrated a reconfigurable polymeric optofluidic compound eye imaging system with nine ommatidium. Each fluidic ommatidium can vary its radius of curvature for a monocular depth sensation. It had a viewing angle of about 120° and a focusing power ranging from about 0 D to 275 D [9]. Zeng et al. have demonstrated a biologically artificial compound eye that has a wide FOV and a tunable focal length. The system consisted of an array of liquid micro-lenses enclosed in compliant membranes, and the liquid micro-lenses could tune their focal length from 64.3 to less than 2.1 mm [10,11]. Ali Shahini et al. have demonstrated a compound eye imaging system with nine ommatidia made of ionic liquid lenses, of which both curvatures can be individually changed using electrowetting on dielectric and applied pressure. The ommatidium can tune the focal length from 3.55 mm to 3.70 mm [12,13,14,15]. However, tunable artificial compound eye imaging systems using liquid lenses suffer from several inevitable problems because of the intrinsic characteristics of the liquid material, including gravity effect, shape instability, evaporation, and temperature sensitivity. Those tunable artificial compound eye imaging systems using liquid crystal lenses are polarization-dependent and have a small aperture. Moreover, an optical zoom imaging system that can vary the magnification factor without displacing the object or the image plane has shown great potential in a broad array of applications. Although these tunable artificial compound eye imaging systems are capable of tunable the focal length of ommatidia, it is difficult to maintain the image plane for continuous optical zoom. The movement of the image sensor is needed to obtain a clear image. Reference [16] proposed a continuous zoom compound eye imaging system based on seven liquid lenses without any mechanical movement of imaging components. However, the liquid lenses are sensitive to gravity and temperature, and the actuators of these liquid lenses are pumps, which makes the response speed slow.

Different from the liquid lens and liquid crystal lens, the Alvarez lens is a novel kind of adaptive lens [17,18]. Due to the lack of liquid material and liquid crystal involved, the Alvarez lenses show several remarkable advantages in terms of temperature sensitivity, response speed, and environmental stability. Moreover, the Alvarez lens is composed of two phase plates, which have complementary cubic surface profiles. By slightly shifting two phase plates relative to each other perpendicular to the optical axis, a variation in optical power can be achieved. The Alvarez lens can provide a wide focal length tuning range. Therefore, the Alvarez lens is suitable for developing a continuous optical zoom compound eye imaging system. However, the existing actuators to generate the lateral shifting for the Alvarez lens include MEMS-driven units [19,20], motors [21], artificial muscles [22], piezoelectric materials [23,24], and manual operations [25]. However, these actuators have either a small stroke and a complex structure or suffer from a slow response speed. It is difficult to realize continuous optical zoom compound eye imaging with large magnification and high response speed. The dielectric elastomer (DE) is a remarkable active actuator that is well known as an “artificial muscle”. It is rapidly becoming a preferred choice of soft actuators because it can achieve large actuated strain, quick response speed, lightweight, low cost, and the absence of noise pollution [26,27,28]. In this manuscript, we study the continuous optical zoom compound eye actuated by the dielectric elastomer.

To provide an alternative for tunable artificial compound eye imaging [29], a continuous optical zoom compound eye imaging system based on the Alvarez lenses is proposed. As a new exploration, we use Alvarez lenses as the ommatidia of the compound eye, so that the ommatidia have focal length adjustment ability. To the best of our knowledge, optical zoom compound eye imaging using Alvarez lenses actuated by DEs is demonstrated for the first time. Compared with other works on artificial compound eyes, there are two key novelties of our proposed system. Firstly, the refractive elements of the ommatidia in the proposed compound eye imaging system are all Alvarez lenses, which have no liquid involved, resulting in ease of packaging and handling with no potential leakage and evaporation issues and thereby making it more mechanically and environmentally robust. Secondly, all Alvarez lenses in the proposed continuous optical zoom compound eye imaging are actuated by DEs. By applying voltages to the DEs for actuating these Alvarez lenses, the focal lengths and the magnification of the optical zoom compound eye imaging system can be altered. The simulated and experimental results show that the proposed compound eye imaging system is capable of providing a 3× continuous optical zoom, making it applicable to observing more object’s details. The rest of the paper is organized as follows: Section 2 describes the principle of the proposed continuous optical zoom compound eye imaging system using Alvarez lenses actuated by DEs. Section 3 presents the optical simulation, and Section 4 presents the design and fabrication process of the proposed continuous optical zoom imaging system. The experimental results and discussion are presented in Section 5. Section 6 is the conclusion.

## 2. Principle of the Proposed Continuous Optical Zoom Compound Eye Imaging System

**Figure 1:**
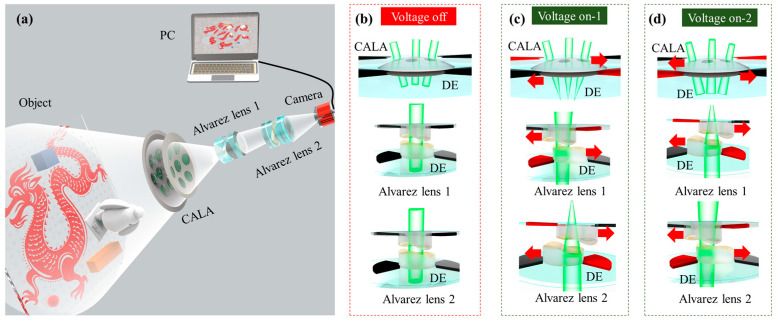
(**a**) The schematic of the proposed continuous optical zoom compound eye imaging system. (**b**) These phase plates are precisely aligned with each other along the optic axis when the actuation voltage is off. (**c**) By applying driving voltages to different fan-shaped compliant electrodes, the CALA and the two Alvarez lenses can act as a convex lens or a concave lens. The CALA acts as a convex lens, the Alvarez lens 1 acts as a concave lens, and the Alvarez lens 2 acts as a convex lens. (**d**) The CALA acts as a concave lens, and the Alvarez lens 1 acting as a convex lens, as well as the Alvarez lens 2 acts as a concave lens.

The optical schematic layout of the proposed continuous optical zoom compound eye imaging system using Alvarez lenses actuated by DEs is shown in Figure 1. The main elements consist of a curved Alvarez lens array (CALA), two Alvarez lenses (Alvarez lens 1 and Alvarez lens 2), and a camera. The CALA includes seven Alvarez lenses, and each Alvarez lens of the CALA is composed of two-phase plates. The phase plate array is arranged on a shell with a curved surface to mimic the large FOV feature of the insect compound eye. Afterward, two Alvarez lenses form the optical structure of the Keplerian telescope. A camera with an objective is used to capture images. The focal lengths of the CALA and the two Alvarez lenses can be independently changed to realize continuous optical zoom compound eye imaging.

The two-phase plate arrays of the CALA are mounted on the surfaces of two DEs, respectively. Similarly, the two-phase plates of the two Alvarez lenses are also mounted on the surface of two DEs, respectively. Both sides of two fan-shaped areas of the DEs around these phase plates are coated with compliant electrodes. The relative movement of these phase plates perpendicular to the optical axis is used to change the focal lengths of the CALA and the two Alvarez lenses. The movement is realized by the actuation of the DEs. Initially, i.e., when the actuation voltage is off, these phase plates are precisely aligned with each other along the optic axis, as shown in Figure 1b. When the actuation voltage is on, electrostatic attraction of the DE, i.e., Maxwell’s stress, produces radially compressive force directed toward the phase plate at the center and subsequently pulls the phase plate to move laterally towards the edge. Therefore, the focal lengths of the CALA and the two Alvarez lenses can be tuned. By applying driving voltages to different fan-shaped compliant electrodes, two phase plates can be moved in different directions. Therefore, the focal length of the CALA and the two Alvarez lenses can not only be changed by controlling the lateral displacement of the phase plates but can also be varied from a convex lens to a concave lens. As shown in Figure 1c, the CALA acts as a convex lens, the Alvarez lens 1 acts as a concave lens, as well as the Alvarez lens 2 acts as a convex lens. Similarly, as shown in Figure 1d, the CALA acts as a concave lens, and the Alvarez lens 1 acts as a convex lens, and the Alvarez lens 2 acts as a concave lens. Therefore, by adjusting the actuation voltage for the DEs, the proposed system can realize continuous optical zoom imaging. In Figure 1c,d, the red arrow indicates the direction in which the CALA and Alvarez lenses are moving.

The CALA and two Alvarez lenses are composed of two-phase plates. Each phase plate has a planar-freeform shape, i.e., one plane surface side and one free-form surface side described by a cubic polynomial equation. The profiles of the two-phase plates can be described by the following equations [17,18]

(1) $$t_{1}=A(xy^{2}+x^{3}/3)+Dx+E,$$

(2) $$t_{2}=-A(xy^{2}+x^{3}/3)-Dx+E,$$

where *A*, *D*, and *E* are constants to be determined as well as *x*, and *y* are transverse coordinates normal to the z-direction, and *t*_1_ and *t*_2_ are the phase profiles of the two-phase plates. An amplitude coefficient *A* controls the amount of free-form surface depth modulation over a given area. The constant *D* defines the tilt of the free-form surface and can be used to reduce the overall thickness of the phase plates. The constant *E* is the bulk element thickness added to the cubic surface equation at the center. The focal lengths of the CALA and two Alvarez lenses can be changed by controlling the lateral displacement of the phase plates, which can be expressed by [30,31]

(3) $$f=\frac{1}{4\delta A(n-1)},$$

where *f* is the focal length, *δ* is the lateral displacement of the phase plates, and *n* is the refractive index of the phase plate material.

**Figure 2:**
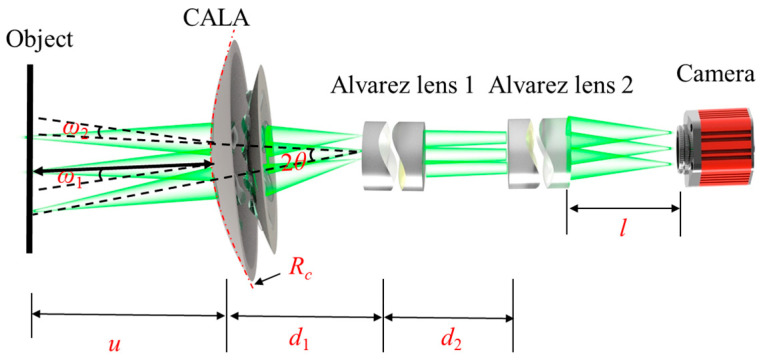
Schematic diagram of the proposed system’s optical path.

The schematic diagram of the proposed system’s optical path is shown in Figure 2. The object is illuminated by a uniform white LED light source. A virtual relay image is formed between the CALA and the Alvarez lens 1 by the CALA. Then, the image is transferred to the camera by the Alvarez lens 1 and Alvarez lens 2. According to the theory of paraxial optics, the focal power Φ of the central optical channel of the proposed imaging system can be expressed by

(4) $$\Phi =\Phi_{c}+\Phi_{1}+\Phi_{2}-d_{1}\Phi_{c}\Phi_{1}-d_{2}\Phi_{1}\Phi_{2}+d_{1}d_{2}\Phi_{c}\Phi_{1}\Phi_{2},$$

where Φ*_c_* is the focal power of the CALA, Φ_1_ is the focal power of the Alvarez lens 1, Φ_2_ is the focal power of the Alvarez lens 2, *d*_1_ is the central distance between the CALA and the Alvarez lens 1, and *d*_2_ is the central distance between the Alvarez lens 1 and the Alvarez lens 2. When the working distance (*u*) and back working distance (*l*) are fixed, the focal powers of the CALA and two Alvarez lenses, i.e., Φ*_c_*, Φ_1_, and Φ_2,_ can be changed to achieve continuous variable paraxial magnifications of the proposed optical zoom compound eye imaging system.

The theoretical value of the FOV of the proposed continuous optical zoom compound eye imaging system can be calculated by

(5) $$\mathrm{FOV}=2\;\arcsin\frac{2H\cdot r_{s}}{r_{s}^{2}+H^{2}},$$

where *H* and *r_s_* are the height and the radius of the curved surface, respectively. Because the CALA contains seven Alvarez lenses, the FOV of adjacent Alvarez lenses should overlap to avoid loss of object information, as shown in

(6) $$\omega_{1}(\Phi )+\omega_{2}(\Phi )>2\theta ,$$

where ω_1_ is the half FOV of the central optical channel of the CALA, and ω_2_ is the half FOV of the edge optical channel of the CALA, and *θ* is the angle between the central channel’s and the edge channel’s optical axes.

## 3. Optical Simulation of the Proposed Continuous Optical Zoom Compound Eye Imaging System

**Figure 3:**
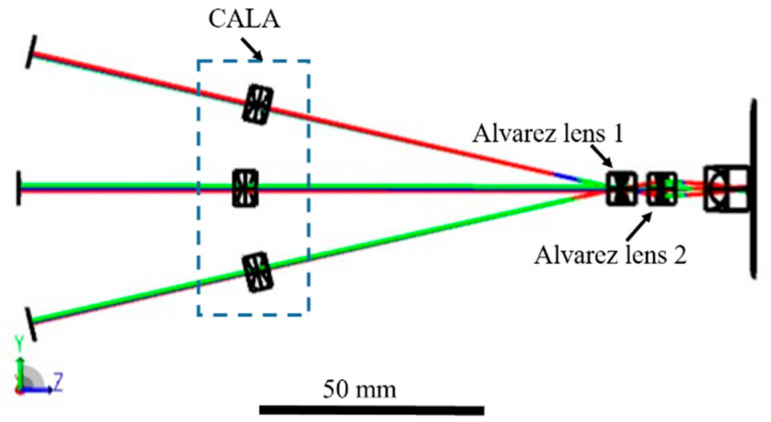
Simulation model of the system’s optical path by the optical simulation software.

To obtain the optical parameters and evaluate the imaging performance, we employ OpticStudio 20.3.2 software [18,30,32] (Figure S1) to carry out the optical simulation of the proposed imaging system. The initial optical structure of the proposed system is shown in Figure 3. The parameters to describe the freeform surface of phase plates are A = 0.075 mm^−2^, D = −0.175, and E = 1 mm in Equations (1) and (2) based on simulation analysis and references [33,34]. The lateral displacement δ of the phase plates of the CALA and two Alvarez lenses is set to variables to optimize the imaging system. The specific surface parameters and the lateral displacements of the phase plates of the proposed imaging system at different magnifications are listed in Table S1.

**Figure 4:**
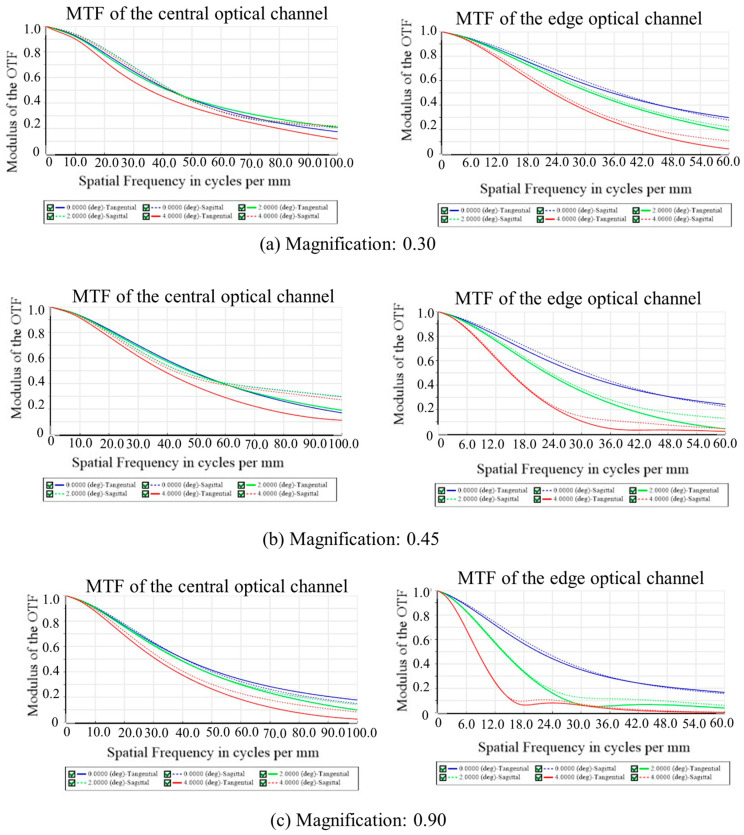
Three-dimensional layout, MTF of the central optical channel, and MTF of the edge optical channel of the proposed imaging system at the paraxial magnifications of (**a**) 0.30, (**b**) 0.45, and (**c**) 0.9.

We simulate continuous zoom imaging at variable paraxial magnifications from 0.30 to 0.90. The working distance is set to be 150 mm, and the back working distance is set to be 16 mm. The size of the image sensor is 2/3”, and the total length of the proposed system is 100 mm. We selected the modulation transfer function (MTF) to evaluate the imaging performance of the proposed imaging system. The 3D Layout, MTF of the central optical channel, and MTF of the edge optical channel at the paraxial magnifications of 0.30, 0.45, and 0.90 are shown in Figure 4a–c. The results show that the value of the MTF decreases with increasing magnification. When the paraxial magnification is 0.30, the MTF of the central optical channel is 0.2@80 lp/mm, and that of the edge optical channel is 0.2@42 lp/mm. When the paraxial magnification is 0.45, the MTF of the central optical channel is 0.2@76 lp/mm, and that of the edge optical channel is 0.2@42 lp/mm. When the paraxial magnification is 0.90, the MTF of the central optical channel is 0.2@60 lp/mm, and that of the edge optical channel is 0.2@14 lp/mm.

**Figure 5:**
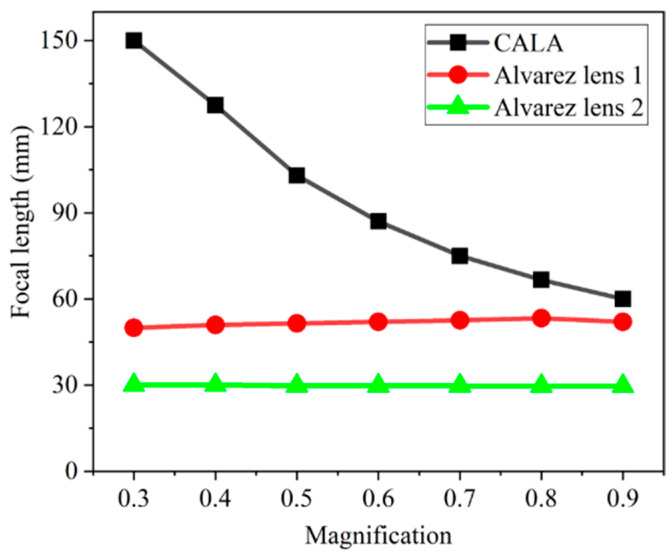
Relationships between the magnifications and the focal lengths of the CALA Alvarez lens 1 and Alvarez lens 2.

By adjusting the lateral displacement of phase plates, the focal length of the CALA Alvarez lens 1, and Alvarez lens 2 can be changed to achieve continuous variable paraxial magnifications of the proposed system. The relationship between the paraxial magnification and the focal length of the CALA, Alvarez lens 1 and Alvarez lens 2 is shown in Figure 5. When the magnification increases from 0.30 to 0.90, the focal length of the CALA decreases from 150 mm to 60 mm, and that of the Alvarez lens 1 increases from 50 mm to 52 mm. The focal length of the Alvarez lens 2 is slightly changed because it is used to correct aberrations.

## 4. Fabrication of a Continuous Optical Zoom Compound Eye Imaging System

**Figure 6:**
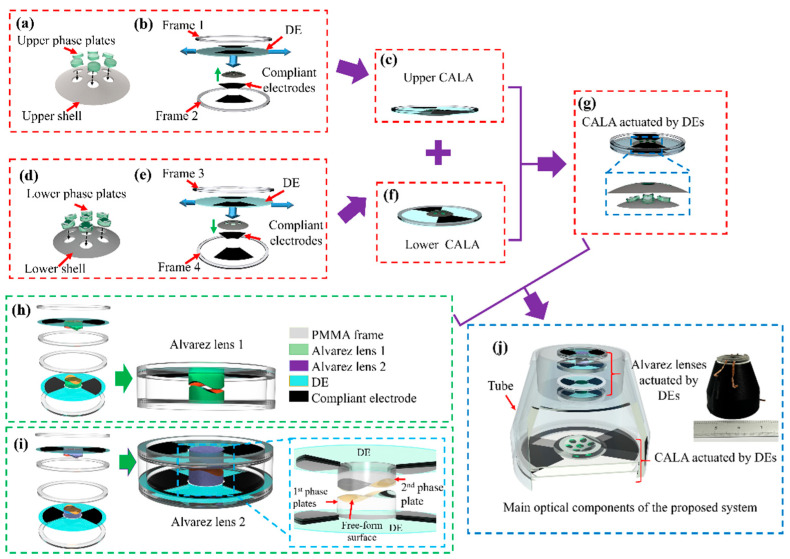
Schematic illustration of the fabrication procedure of the main optical components of the proposed continuous optical zoom compound eye imaging using Alvarez lenses actuated by DEs. (**a**) The upper-phase plates of the CLAL are precisely assembled into the upper shell. (**b**) The DE is sandwiched by the two PMMA frames. The top and bottom sides of fan-shaped areas of the DE are coated with compliant electrodes. The upper-phase plates of the CLAL are precisely mounted on the center area of the DE under a microscope. (**c**) The components are precisely assembled to form the upper CALA. (**d**–**f**) The fabrication procedure of the lower CALA actuated by DE. (**g**) The upper CALA and lower CALA are combined to form the CALA actuated by DEs. (**h**,**i**) The fabrication procedure of two Alvarez lenses actuated by DEs. (**i**) The section view of the main optical components of the proposed continuous optical zoom compound eye imaging using Alvarez lenses actuated by DEs. (**j**) Section view and photograph of the proposed continuous optical zoom compound eye imaging system.

The fabrication process of the proposed continuous optical zoom compound eye imaging using Alvarez lenses actuated by DEs is shown in Figure 6a–j, respectively. The material of the phase plates of the Alvarez lenses is the UV-curable optical adhesive NOA83H (Norland, Jamesburg, NJ, USA) with a refractive index of 1.56. The phase plates of the CALA and the two Alvarez lenses have the same freeform surfaces. The diameter of these phase plates is 6.0 mm, and we fabricate them through the single-point diamond-turning (Nanoform 250, Precitech, Keene, NH, USA) and replication molding processes. The Nanoform 250 has five linear axes that are equipped with a programming resolution of 0.01 nanometers. To fabricate the Alvarez lenses, the workpiece is mounted on the main spindle with its angular position fixed. The movements of three linear axes (X, Y, and Z axis) were simultaneously controlled to preset positions in sequence based on the surface equation. The diamond tool feed rate is 100 mm/min. The tool nose radius of the diamond cutter is 250 lm in this study. The fabrication process of the proposed system is mainly divided into two parts. The first one is the fabrication of the CALA actuated by DEs. An upper shell with a curved surface is fabricated by a 3D printer (Pro3 Plus, Raise3D Company, Yangpu district, Shanghai, China), and seven phase plates of the CALA are accurately embedded in the holes of the upper shell as shown in Figure 6a. A commercial VHB4905 tape (3M Company, St. Paul, MN, USA) is chosen as the DE actuator. The DE is biaxially stretched by a factor of 200% and then sandwiched by the two PMMA frames with an inner diameter of 50 mm and an outer diameter of 54 mm, as shown in Figure 6b. The top and bottom sides of fan-shaped areas of the DE are coated with carbon powder (BP2000, Carbot, Boston, MA, USA) as compliant electrodes by using a brush. The upper seven phase plates of the CALA are precisely mounted on the center area of the DE under a microscope camera, as shown in Figure 6c. The lower CLAL has the same fabrication process as the upper CALA, as shown in Figure 6d–f. The lower CLAL is rotated 180° and is combined with the upper one to form the CALA actuated by DEs, as depicted in Figure 6g. The fabricated the upper and lower part of the CALA is shown in Figure S2. The second one is the fabrication of two Alvarez lenses actuated by DEs. It has the same fabrication processes as the CALA actuated by DEs, as shown in Figure 6h,i. The CALA actuated by DEs and the two Alvarez lenses actuated by DEs are preciously mounted into a 3D printed tube to form the main optical components of the proposed continuous optical zoom compound eye imaging system, which has a dimension of 54 mm × 36 mm ×60 mm (L ×W × H), as shown in Figure 6j.

## 5. Experiments and Discussion

**Figure 7:**
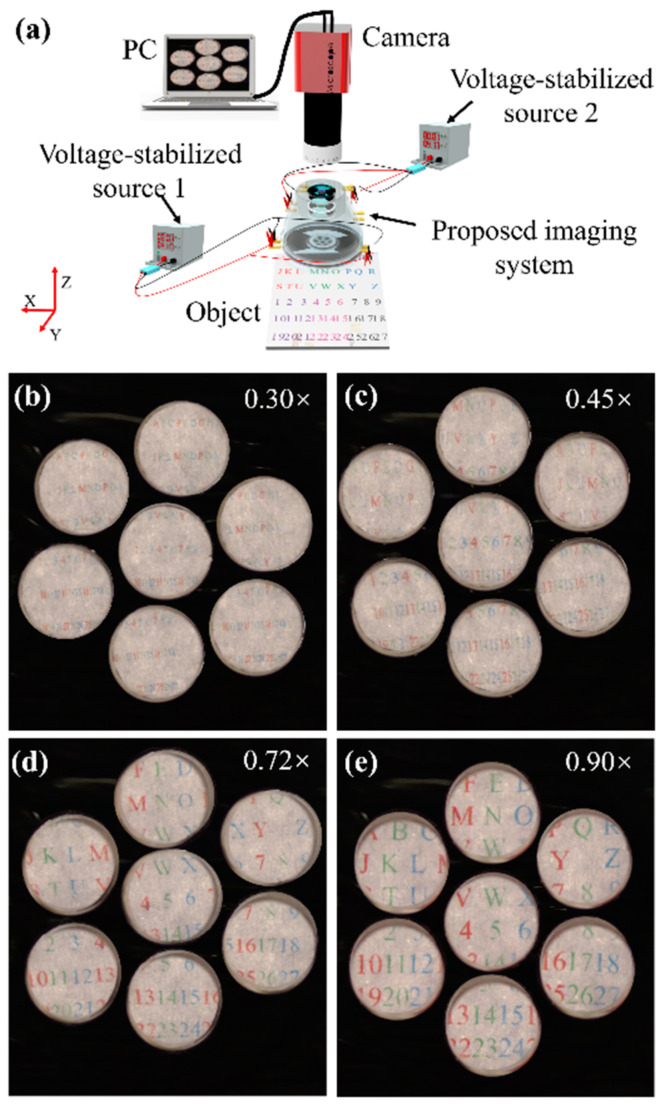
(**a**) Experimental schematic for verifying the optical zoom capabilities of the proposed imaging system. (**b**–**e**) Captured images of the object at different magnifications.

The experimental schematic for measuring the imaging abilities of the proposed imaging system is shown in Figure 7a. Two voltage-stabilized sources (UTP3315TFL-II, UNI-T Company, Dongguan, Guangdong, China) are used to produce actuation voltages. The actuation voltage for the CALA actuated by DEs is generated from voltage-stabilized source 1, and the actuation voltage for Alvarez lens 1 actuated by DEs is generated from voltage-stabilized source 2. The driving voltage is amplified 1200 times by a high-voltage converter (A60P-5, XP-Power Company, Taiseng district, Singapore). A microscope camera (GP-530H, Kunshan Gaopin Precision Instrument Company, Kunshan, Jiangsu, China) is used to capture images. The frame rate of the camera is 60 fps @1920 × 1080, the sensor format is 1/2.5” as well as the exposure time is 16 ms. A board marked with colored letters “A–Z” and numbers “1–27” is regarded as the object and is illuminated by a uniform white light source. The size of the board is 92 mm × 92 mm. The board is placed 200.0 mm away from the proposed imaging system. The distance between the CALA and Alvarez 1 is 40 mm, and the distance between Alvarez 1 and Alvarez 2 is 20 mm, as well as the distance between Alvarez 2 and the camera, which is 300 mm. In Equation (5), H and rs are set to be 5.40 mm and 42.34 mm, respectively. Therefore, the theoretical FOV is calculated to be approximately 29°. The actual FOV is 30° according to the trigonometric function, i.e., 2 × arctan (46 mm/200 mm). The two-phase plates of the Alvarez lens 2 are laterally offset along the x-axis to obtain a focal length of 50 mm. By adjusting the actuation voltage of the two voltage-stabilized sources, the focal length of the CALA and the Alvarez 1 actuated by DEs can be changed, and then the magnification of the proposed imaging system can be tuned. The imaging results are shown in Figure 7b–e. The paraxial magnification is defined as the ratio of the paraxial image height to the object height. The results show that when the voltage-stabilized source 1 generates the actuation voltage from 0 V to 2.5 V and the voltage-stabilized source 2 generates the actuation voltage from 0 V to 1.5 V, the magnification can vary from 0.3× to ∼0.9×. The zoom ratio of the proposed imaging system can reach 3×. Although there are some deviations between the actual zoom range and the simulation data, the experimental results are consistent with the theory. The zoom range can be further expanded, but there is a trade-off between imaging quality and zoom range. In addition, it could be observed that there is an overlap between the FOV of each sub-image.

**Figure 8:**
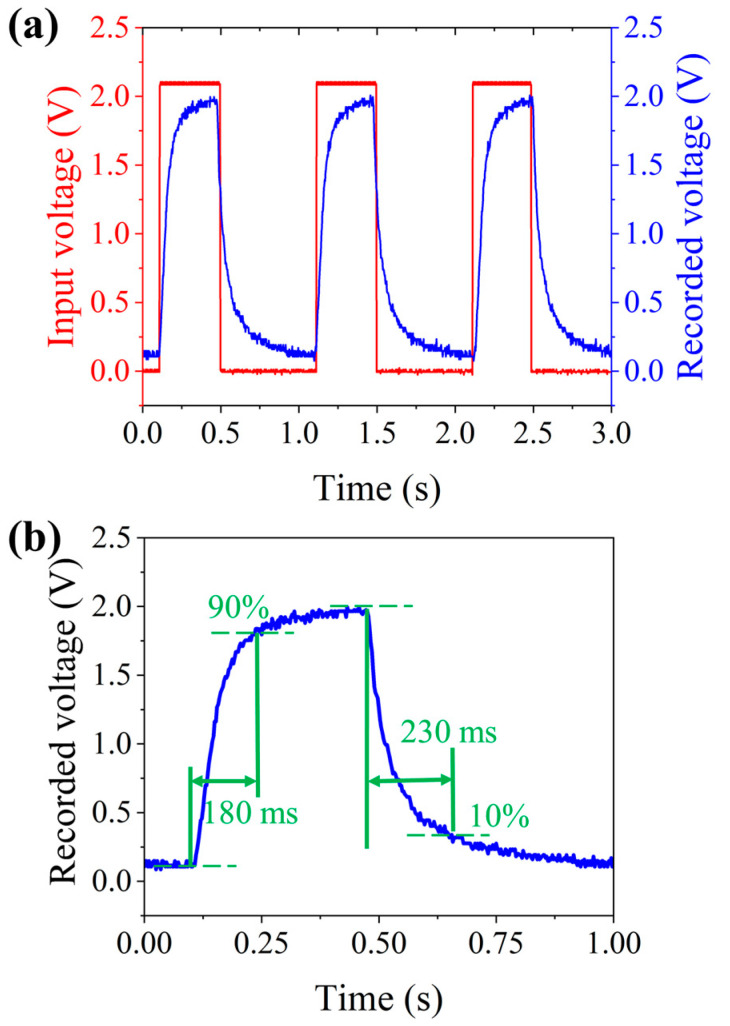
Experimental results to measure the dynamic response time of the proposed imaging system. (**a**) The recorded voltage of the photodetector when the input voltage is a square signal with a frequency of 1Hz, a duty cycle of 50%, and an amplitude of 3.0 V. (**b**) The measured rise time and fall time are 180 ms and 230 ms, respectively.

By adjusting the actuation voltage for the DEs, the proposed system can realize continuous optical zoom imaging. To evaluate the dynamic performance, the response time of the proposed imaging system is tested. A collimated 632 nm laser beam is employed as the light resource to illuminate the proposed system. A photodetector (PDA36A-EC, Thorlabs) is placed at the focal plane of the proposed imaging system and is used to obtain the response voltage signal. A function generator (DG1062, RIGOL Technologies, Suzhou, China) provides a periodical square wave input voltage with a period of 1 s, peak-to-peak amplitude of 3.0 V, and a duty cycle of 50%. The input voltage is amplified by a power amplifier (PA1011, RIGOL Technologies) and then applied to actuate the proposed imaging system. When the input voltage is applied to DEs to actuate the CALA and the two Alvarez lenses, the focal length of the proposed imaging system is changed, and then the voltage recorded by the photodetector is varied. The input voltage and the recorded voltage are presented in Figure 8a. The rise time and fall time are defined as the time consumption from the initially recorded voltage to 90% of the maximum recorded voltage and from the maximum recorded voltage to 10% of the maximum recorded voltage. The rise time and fall time of the proposed imaging system are depicted in Figure 8b. The results show that rise and fall times are 180 ms and 230 ms, respectively.

**Figure 9:**
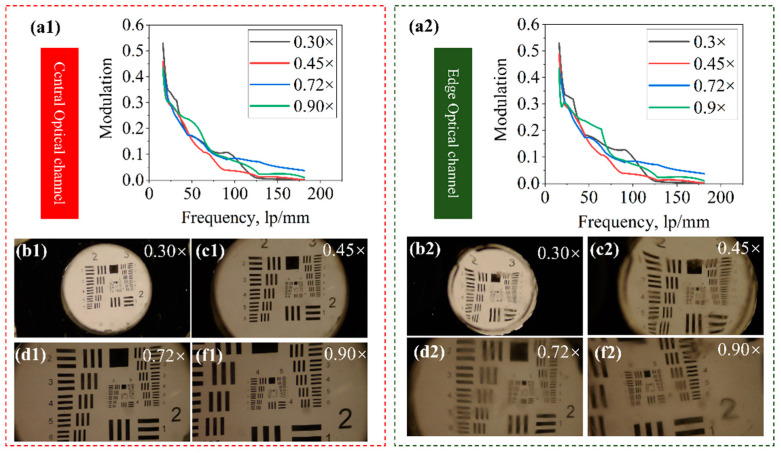
(**a1**) The modulation and frequency relationship of the central optical channel at different magnifications (**a2**) The modulation and frequency relationship of the edge optical channel at different magnifications; (**b1**–**f1**) The captured images of the central optical channel and edge optical channel (**b2**–**f2**) of the USAF 1951 resolution target at different magnifications.

We also measure the MTF curves of the central and edge optical channels of the proposed imaging system to evaluate its optical resolving power. The MTF values are calculated based on the captured images of the USAF 1951 resolution target. The modulation and frequency relationship of the central optical channel and the edge optical channel at different magnifications are presented in Figure 9(a1,a2). The target can be focused by the proposed imaging system and recorded by the image sensor at different magnifications without the movement of the camera. The approach that we utilize to calculate the MTF values is explained in detail in the references [35,36]. The captured images at magnifications of 0.30×, 0.45×, 0.72×and 0.90× are shown in Figure 9(b1–f1,b2–f2). The results show that when the value of MTF is greater than ∼0.1 and magnifications are 0.30, 0.45, 0.72, and 0.9, the spatial frequencies of the central optical channel’s FOV can reach ∼100 lp/mm, 85 lp/mm, 80 lp/mm, and 75 lp/mm, while those of the edge optical channel’s can reach ∼95 lp/mm, 75 lp/mm, 90 lp/mm, and 90 lp/mm, respectively. When the value of MTF is greater than ∼0.2 and magnifications are 0.30, 0.45, 0.72, and 0.9, the spatial frequencies of the central optical channel’s FOV can reach ∼50 lp/mm, 55 lp/mm, 56 lp/mm, and 70 lp/mm, while those of the edge optical channels can reach ∼55 lp/mm, 50 lp/mm, 52 lp/mm, and 75 lp/mm, respectively. The MTF curves vary slightly at different magnifications, and the maximum optical resolution is maintained at around 50 lp/mm during the whole tuning range. It is noted that the experimental result is lower than the simulated one. The reasons include the nonuniform illumination, scattering of the lens elements, and assembly errors.

In summary, tunable artificial compound eye imaging systems using liquid lenses and other tunable lenses suffer from several inevitable problems because of the intrinsic characteristics of the liquid material. Although these tunable artificial compound eye imaging systems are capable of t the focal length of ommatidia, it is difficult to maintain the image plane for continuous optical zoom. As a new exploration, we use Alvarez lenses as the ommatidia of the compound eye, so that the ommatidia have focal length adjustment ability. From Table 1, comparing the proposed compound eye and the reported systems, we can find that our compound eye can realize continuous zoom imaging without any mechanical movement vertically to the optical axis.

In the previous study [37], the DE combined with the Alvarez lens could achieve a wide adjustment range of focal length. The varifocal function is realized by the Alvarez lenses, and the scanning function of this element is realized by the scanning elements. In its initial state, the captured image is clear. By applying different voltages, the captured image becomes blurred, and the microscope needs to be re-adjusted to make the image clear again. The implementation and actuation methods of this paper resemble those of the previous publication. However, the principle between the previous study and this paper is different. Although the focal length of the Alvarez lenses is changed by the actuation of the DE, the imaging system in the previous study was a varifocal scanning system rather than a zooming system. In this paper, the proposed compound eye imaging system is a zoom system. The main purpose is to provide the ommatidia with adjustability and maintain the image plane for continuous optical zoom compound imaging. The compound eye imaging system has the merits of a wide FOV, high sensitivity, and detection of moving targets and has aroused extensive concern. We have innovatively combined the Alvarez lens and dielectric elastomer into continuous optical zoom compound eye imaging systems to provide an alternative for tunable artificial compound eye imaging. A continuous optical zoom compound eye imaging system based on the Alvarez lenses is proposed. As a new exploration, we use Alvarez lenses as the ommatidia of the compound eye, so that the ommatidia have focal length adjustment ability. By adjusting the applied voltages to the DE, the Alvarez lenses are moved laterally, and then the focal lengths of CALA and the two Alvarez lenses are changed. By adjusting the focal lengths of these two Alvarez lenses, the optical magnification is tuned. The microscope does not need to be re-adjusted to make the image clear again. 

In this paper, a continuous optical zoom compound eye imaging system based on the Alvarez lenses actuated by DEs is proposed. The paraxial magnification can range from 0.30× to 0.90× with a response time of 180 ms, which indicates the zoom ratio can reach up to 3.0. The resolution of the central optical channel and edge optical channel of the proposed imaging system can reach 50 lp/mm during the optical zoom process. The proposed imaging system also has some drawbacks, such as

1. The response times are determined by the viscoelastic properties of the DE material, the weight of the Alvarez lenses, and the actuation mechanism. The rise time and fall time of the proposed imaging system are 180 ms and 230 ms, respectively. Those values are not astonishingly greater than our previous studies [29,38,39]. The response time can be further decreased using the Alvarez lens and shell with low density and the DE with a high Young’s modulus.
2. The imaging performance of the system needs to be further improved. The resolution of the central optical channel and edge optical channel of the proposed imaging system is 50 lp/mm during the optical zoom process. The reasons include scattering of the Alvarez lenses and assembly errors. More Alvarez lenses not only increase cost but also make aligning the Alvarez lens more difficult. The further study of our work is to make the Alvarez array into a whole through the single-point diamond cutting method to avoid tolerance buildups owing to combining many Alvarez lenses. Moreover, the imaging performance of the system can be improved by adding the high-order term coefficients of the free-form surface of the Alvarez lenses.

## 6. Conclusions

In this paper, we propose a continuous optical zoom compound eye imaging system based on Alvarez lenses actuated by DEs. The main optical imaging part of the proposed imaging system consists of a CALA and two Alvarez lenses. The phase plates of the CALA and two Alvarez lenses are laterally moved by the actuation of the DEs. By adjusting the actuation voltages to the DEs, the focal length of the CALA and the two Alvarez lenses is tuned, and then the proposed imaging system can realize continuous optical zoom imaging. The paraxial magnification can range from 0.30× to 0.90× with a response time of 180 ms, which indicates the zoom ratio can reach up to 3.0. The experiments demonstrate that with a change in magnification, the resolution of the central optical channel and edge optical channel of the proposed imaging system can reach 50 lp/mm during the optical zoom process. Such a continuous optical imaging system demonstrates the distinct advantages of a wide zoom ratio, compact size, and fast response. The proposed imaging system can find usage in many application fields, such as medical and industrial imaging, machine vision, and distance detection.

## Figures and Tables

**Table 1:** Comparison of reported tunable compound eye imaging systems and the proposed compound eye imaging system.

Reference	Zoom Ratio	Ommatidia Materials	FOV(°)	Ommatidia Focal Length (mm)	Response Time (ms)	Actuation
[9]	-	Liquid lens	120	3.36~+∞	36,000	Syringe pump
[14]	-	Ionic liquid lenses	120	3.55~3.75	150	EWOD
[15]	4	Graphene nanosheets lenslets	160	0.127~0.510	>30,000	Photothermal conversion of graphene nanosheets
[16]	2	Liquid lens	<30	10.7~22.5	<260	Liquid pump
Ours	3	Alvarez lens	30	6~150	180	Dielectric elastomer

‘-’ indicates unknown.

## Data Availability

Data are available upon request due to privacy and ethical restrictions.

## Supplementary Materials

The following supporting information can be downloaded at: https://www.mdpi.com/article/10.3390/biomimetics9060374/s1, Figure S1: The 3D layout of the proposed imaging system at the paraxial magnification of (a) 0.30, (b) 0.45, and (c) 0.9; Figure S2: The photo o the fabricated CALA. (a) The upper part of the CALA. (b) lower part of the CALA. Table S1: Surface parameters lateral displacements of the phase plates.
