# Supplementary material for: Continuous Optical Zoom Compound Eye Imaging Using Alvarez Lenses Actuated by Dielectric Elastomers
Source: Biomimetics (Basel). 2024 Jun 20;9(6):374. doi: 10.3390/biomimetics9060374 (PMC11202164; doi:10.3390/biomimetics9060374)
Supplement: Supplementary file 1 [file biomimetics-09-00374-s001.zip › supplemental-document-5-21.docx]

Continuous optical zoom compound eye imaging using Alverez lenses actuated by dielectric elastomers: supplemental document

**S1. Optical simulation of the proposed continuous optical zoom compound eye imaging system**

To obtain the optical parameters and evaluate the imaging performance, we employ OpticStudio software to carry out the optical simulation of the proposed imaging system. The parameters to describe the freeform surface of phase plates are A=0.075 mm-2, D=-0.175, and E= 1 mm [1-3]. The lateral displacement (*δ*) of phase plates of the CALA and two Alvarez lenses are set to variables to optimize the proposed imaging system. The 3D layouts of the proposed imaging system at different paraxial magnifications are shown in Fig.S1


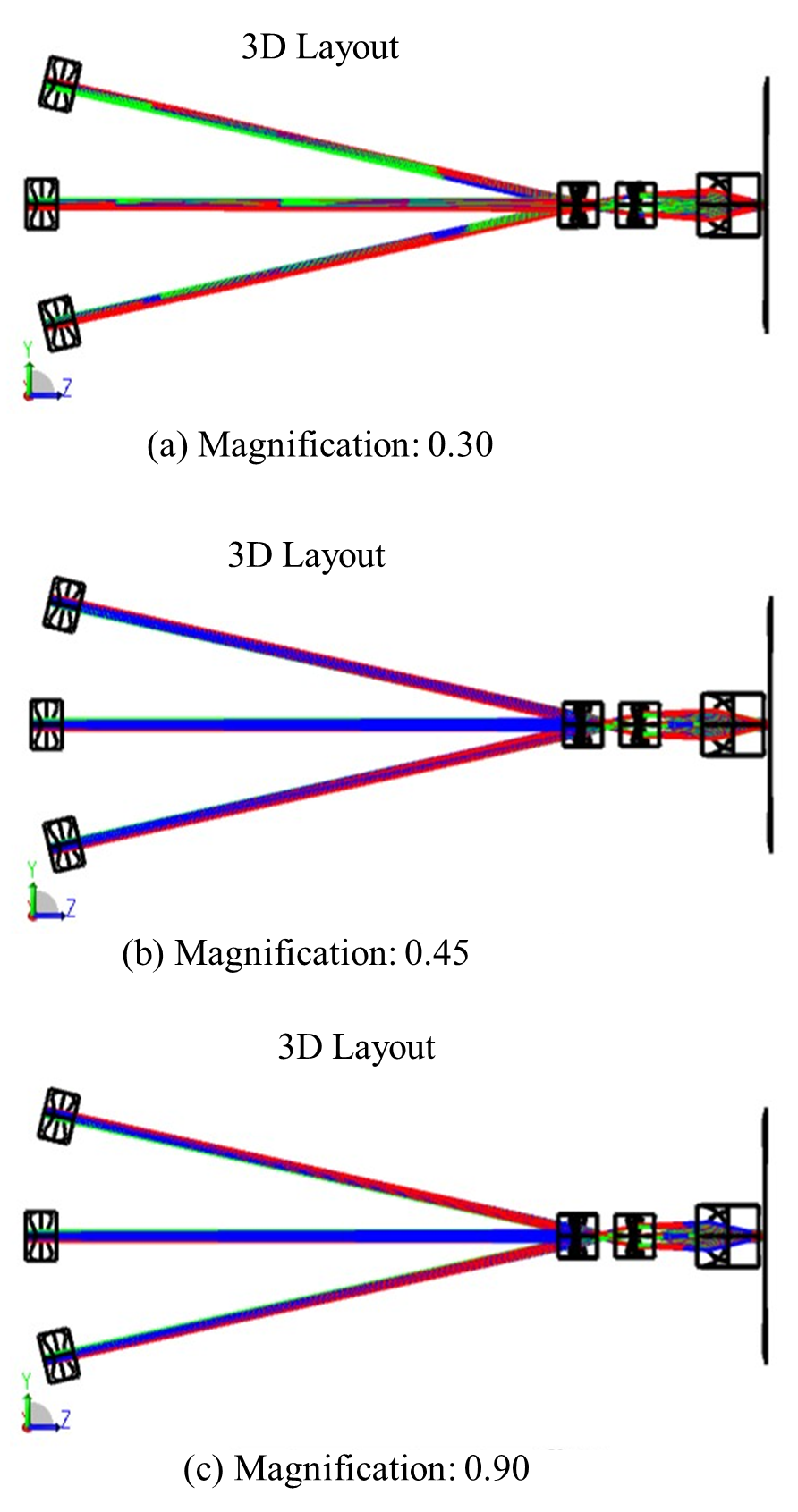


**Figure S1.** The 3D layout of the proposed imaging system at the paraxial magnification of (a) 0.30, (b) 0.45, and (c) 0.9.

The specific surface parameters and the lateral displacements of the phase plates of the proposed imaging system at different magnifications are listed in Table S1.

**Table S1.** Surface parameters lateral displacements of the phase plates

| **Surface number** | **Radius (mm) / Polynomial** | **Material** | **Thickness (mm)** | **Lateral displacement (*δ*) at different magnifications (mm)** | | |
| --- | --- | --- | --- | --- | --- | --- |
|  |  |  |  | **0.3×** | **0.45×** | **0.9×** |
| 1 | Infinity | PMMA | 2 |  |  |  |
| 2 | 0.075(*xy*^2^+*x*^3^/3)-0.175*x* |  | 0.3 | 0.04 | 0.05 | 0.11 |
| 3 | -0.075(*xy*^2^+x^3^/3)+0.175*x* | PMMA | 2 | -0.04 | -0.05 | -0.11 |
| 4 | Infinity |  | 80 |  |  |  |
| 5 | Infinity | PMMA | 2 |  |  |  |
| 6 | 0.075(*xy*^2^+*x*^3^/3)-0.175*x* |  | 0.3 | 0.14 | 0.12 | 0.11 |
| 7 | -0.075(*xy*^2^+*x*^3^/3)+0.175*x* | PMMA | 2 | -0.14 | -0.12 | -0.11 |
| 8 | Infinity |  | 3 |  |  |  |
| 9 | Infinity | PMMA | 2 |  |  |  |
| 10 | 0.075(*xy*^2^+*x*^3^/3)-0.175*x* |  | 0.3 | 0.23 | 0.23 | 0.24 |
| 11 | -0.075(*xy*^2^+*x*^3^/3)+0.175*x* | PMMA | 2 | -0.23 | -0.23 | -0.24 |
| 12 | Infinity |  | 3 |  |  |  |
| 13 | 4 | F2 | 4 |  |  |  |
| 14 | -4 |  | 1 |  |  |  |
| 15 | -5 | F2 | 4 |  |  |  |
| 16 | 5 |  |  |  |  |  |

**S2. Fabricated CALA**

An upper shell with a curved surface is fabricated by a 3D printer (Pro3 Plus, Raise3D Company), and seven phase plates of the upper part of the CALA are accurately embedded in the holes of the upper shell as shown in Fig. S2(a). The lower part of the CALA has the same fabrication process as the upper CALA. The fabricated lower part of the CALA is shown in Fig. S2(b).


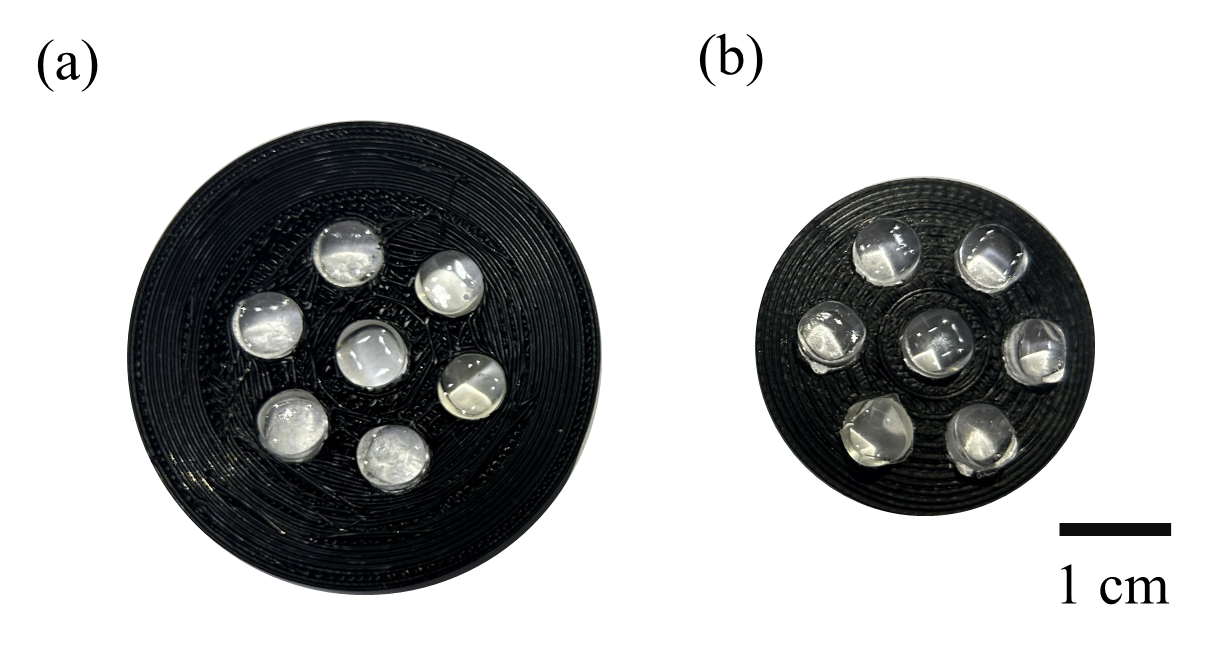


**Figure S2.** The photo of the fabricated CALA. (a) The upper part of the CALA. (b) lower part of the CALA.

References

1. L. Alvarez, "Two-element variable-power spherical lens," (1967).
2. A.W.Lohmann, "A new class of varifocal lenses," APPLIED OPTICS **9**, 1669-1671 (1970).
3. L. A. W. Humphrey, "VARIABLE-POWER LENS AND SYSTEM," (1970).
